# Quasi-Living Polymerization of Propene with an Isotactic-Specific Zirconocene Catalyst

**DOI:** 10.3390/molecules22050725

**Published:** 2017-05-02

**Authors:** Kei Nishii, Miyuki Murase, Takeshi Shiono

**Affiliations:** 1 Department of Materials Chemistry and Bioengineering, National Institute of Technology, Oyama College, 771 Nakakuki, Oyama, Tochigi 323-0806, Japan; k.nishii@oyama-ct.ac.jp; 2 Japan Polypropylene Corporation Polymerization Technical Center 1 Toho-cho, Yokkaichi, Mie 510-0848, Japan; Murase.Miyuki@mb.japanpp.co.jp; 3 Department of Applied Chemistry, Graduate School of Engineering, Hiroshima University, Higashi-Hiroshima 739-8527, Japan

**Keywords:** isotactic, 1-octene, polymerization, propene, quasi-living, zirconocene

## Abstract

Propene polymerization with isotactic (*iso*)-specific *C*_2_-symmetric *rac*-Me_2_Si(2-Me-Benz(e)-Ind)_2_ZrCl_2_ (**1**) and *rac*-Me_2_Si(2-Me-4-Ph-1-Ind)_2_ZrCl_2_ (**2**) were conducted under various conditions for achieving *iso*-specific living polymerization of propene. When Complex **1** was activated with trialkylaluminum-free modified methylaluminoxane (dMMAO) at −40 °C, the number-average molecular weight (*M*_n_) linearly increased against the polymerization time to reach *M*_n_ = 704,000 within 15 min of polymerization, although the molecular weight distributions was broad (*M*_w_/*M*_n_ < 3). Thus, it was found that quasi-living polymerization of propene proceeded in the **1**-dMMAO system. The living nature of *iso*-polypropene was confirmed by the block copolymerization, where the *M*_n_ value increased from 221,000 to 382,000 after the addition of 1-octene to yield the block copolymer with a melting point of 150 °C.

## 1. Introduction

Polyolefins are widely used because of their good chemical and physical properties as well as inexpensiveness and light weight. Especially, isotactic-polypropene (*iso*-PP) has excellent mechanical properties due to its stereoregularity. Recent advances in metallocene catalyst for olefin polymerization allow unprecedented access to new and useful polyolefin architectures [1,2]. However, the economical preparation of olefin block copolymers with the long chain *iso*-PP sequence remains one of the challenges in the field of polymerization catalysis. Such polymers are obtained by block copolymerization with *iso*-specific living polymerization catalysts.

Living polymerization enables us to produce block copolymers by switching the monomer from one to the other during the polymerization [3,4,5,6,7,8]. The simplest and most widely studied type of block copolymer is diblock, where two building blocks of different natures are joined together by a single covalent bond. In the past decade, several noteworthy systems were introduced that led to the living and *iso*-specific polymerization and copolymerization of α-olefins [2,3,4,5]. For example, Coates et al. reported that the pyridylamidohafnium catalyst system produced *iso*-PP-*block*-PE (polyethylene) diblock and multiblock copolymers with precise control of block length (*M*_n_ = 208,000, *mmmm* = 91%, *T*_m_ = 133 °C) [8]. Busico et al. reported the preparation of *iso*-PP-*block*-EPR (poly(ethene-*co*-propene)) using Kol's diamino bis(phenolate)zirconium (or hafunium) catalyst under “quasi-living” conditions (*M*_n_ = 216,000, *mmmm* = 97% *T*_m_ = 143 °C) [9,10]. Sita et al. utilized the living polymerization with (monocyclopentadienyl)zirconium(acetamidinate) catalyst to generate a number of *iso*-PP-containing block copolymers (stereoblock copolymer; *M*_n_ = 172,400, *mmmm* = 71%, *T*_m_ = 115 °C) [6,11,12]. The activity, melting point (*T*_m_), and isotacticity of produced *iso*-PP and *iso*-PP-based block copolymers in these living systems were, however, lower than those of common non-living systems [13,14,15,16,17,18,19]. While there are numerous catalysts for *iso*-specific polymerization and block copolymerization of propene, highly active catalysts for both the living and highly *iso*-specific polymerization and block copolymerization of propene with higher α-olefins remain elusive.

We have previously reported that Me_2_Si(η^1^-N-*^t^*Bu)(η^3^-fluorenyl-derivative)TiMe_2_, [ArN(CH_2_)_3_NAr]TiMe_2_ (Ar = 2,6-*^i^*Pr_2_C_6_H_3_), and Me_2_Si(η^1^-N-*^t^*Bu)(η^5^-C_5_Me_4_)TiMe_2_ activated with trialkylaluminum-free modified methylaluminoxane (dMMAO) in toluene or heptane produced syndiotactic PP and atactic PP at 0 °C and/or 25 °C in a living [20,21,22,23] and quasi-living [24] manner, respectively. On the other hand, in the case of propene polymerization by Me_2_Si(η^1^-N-*^t^*Bu)(η^4^-indenyl)TiMe_2_ activated with dMMAO in heptane at 0 °C, deactivation occurred [25]. These results suggest that the living character strongly depended on the combination of the organometallic complexes, activator, and solvent as well as the polymerization temperature. In this paper, we carried out propene polymerization with *C*_2_-symmetric zirconocene catalysts and found that *rac*-Me_2_Si(2-Me-Benz(e)-Ind)_2_ZrCl_2_ (**1**) activated by dMMAO conducted “quasi-living” and “highly *iso*-specific” polymerization in a high activity at −40 °C. Block copolymerization of propene and 1-octene was also achieved to give *iso*-PP-*block*-poly(propene-*co*-1-octene) by the sequential addition of the monomers.

## 2. Results and Discussion

### 2.1. Polymerization of Propene

Catalytic ability of *C*_2_-symmetric *rac*-Me_2_Si(2-Me-Benz(e)-Ind)_2_ZrCl_2_ (**1**) and *rac*-Me_2_Si(2-Me-4-Ph-1-Ind)_2_ZrCl_2_ (**2**) for propene polymerization was investigated in toluene at 0 °C under an atmospheric pressure of propene using dMMAO as a cocatalyst with an [Al]/[Zr] ratio of 800 and 400. The results are shown in Table 1 (Runs 1–4). Because of the ineffective stirring caused by the produced polymer, the polymerization stopped in 2 min with **1** and 5 min with **2**, respectively. Complex **1** showed approximately 2 times higher activity than Complex **2** regardless of the [Al]/[Zr] ratio. Complex **1** produced higher molecular weight polymers than Complex **2** in spite of the shorter polymerization time. These results indicate the superior catalytic ability of **1** under these polymerization conditions.

On the basis of the results described above, we chose the **1**-dMMAO system to examine the effects of polymerization conditions in more detail. The **1**-dMMAO system showed very high activities with the [Al]/[Zr] ratio of 400 and 800, which prevented us from evaluating the catalytic ability correctly. We therefore reduced the [Al]/[Zr] ratio to 300 and 100 (Runs 5 and 7). The catalytic performance with [Al]/[Zr] = 300 was almost the same with those with [Al]/[Zr] = 400 and 800. When the [Al]/[Zr] ratio was reduced to 100, the activity decrease to approximately one-twentieth, whereas the *M*_n_ value of the produced polymer increased from approximately 200,000 to 366,000. Consequently, the number of polymer chains per Zr used (*N*) decreased by one-fiftieth. These results implied that the low polymerization activity with [Al]/[Zr] = 100 was caused by the low initiation efficiency. Almost the same activities above, [Al]/[Zr] = 300 should have been caused by the dissolving limitation of gaseous propene to the solvent, which yielded low *M*_n_ values compared with [Al]/[Zr] = 100.

One of the characteristics of dMMAO is a good solubility in saturated hydrocarbon solvents. Therefore, the polymerization was carried out in heptane at a [Al]/[Zr] of 300 (Run 6). The catalytic activity was decreased from 5000 kg-PP·mol-Zr^−1^·h^−1^ in toluene to 300 kg-PP·mol-Zr^−1^·h^−1^ in heptane. The *M*_n_ value of the produced polymer increased from 238,000 in toluene for 2 min polymerization to 449,000 in heptane for 10 min polymerization. The *N* value decreased from 0.70 in toluene to 0.11 in heptane. On the assumption that chain transfer reactions are suppressed, these results imply higher propagation rate and higher initiation efficiency in toluene than in heptane. The former can be explained by the separation of the active Zr cation and the dMMAO-derived anion in the polar solvent. We [22] and Fink et al. [26] reported the relationship between the polarity of the solvent and the polymerization rate. The low initiation efficiency can be explained by the higher solubility of **1** in toluene than in heptane.

In order to investigate the livingness of the **1**-dMMAO system in heptane, we conducted batch-wise polymerizations of propene at 0 °C for one hour by changing the amount of propene in feed. The results are shown in Table 2. The polymerization proceeded quantitatively (yield: 91–93%) regardless of the charged propene amount. Furthermore, we conducted post-polymerization where the same amount of monomer was sequentially added after the first polymerization had been completed. Although the post-polymerizations proceeded quantitatively (yield > 90%), the *M*_n_ values only slightly decreased (1/1.23 times) with keeping the *M*_w_/*M*_n_ values almost constant. These results indicate that deactivation did not occur, but chain transfer reactions occurred in the polymerization at 0 °C within one hour.

**Figure 1:**
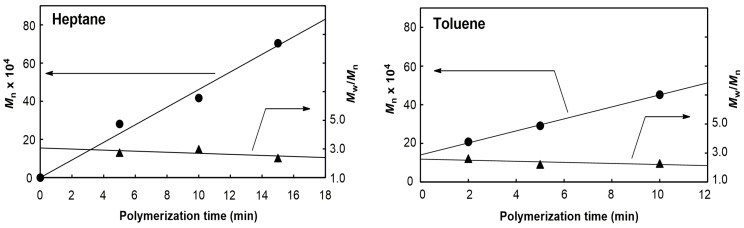
Plots of *M*_n_ and *M*_w_/*M*_n_ as a function of the polymerization time for polypropenes prepared at −40 °C with **1**-dMMAO ([Zr] = 5 μmol; [Al]/[Zr] = 600; propene = 1 atm).

To suppress the chain transfer reactions, we conducted polymerization at −40 °C in toluene and heptane, and investigated the time dependence of *M*_n_ by sampling the polymer during polymerization. The results are summarized in Table 3. The *M*_n_ and *M*_w_/*M*_n_ values thus obtained were plotted against the polymerization time in Figure 1, which shows a good linear relationship. However, in the toluene system, the straight line did not go through the origin. The phenomenon should be attributed to the change of the monomer concentration because of the higher activity in toluene as shown in Table 1. Although *M*_w_/*M*_n_ was broad (*M*_w_/*M*_n_ < 3), it became narrow as the polymerization time increased, suggesting no chain transfer and termination reactions. These results indicate that the **1**-dMMAO system promoted quasi-living polymerization of propene at −40 °C.

### 2.2. Block Copolymerization of Propene and 1-Octene

Block copolymerization of propene and 1-octene was conducted with **1**-dMMAO at −40 °C in toluene. After 2 min of propene homo-polymerization followed by sampling the prepolymer, a prescribed amount of 1-octene was added, and copolymerization was conducted for 10 min. The results are summarized in Table 4. The polymers obtained after the 1-octene addition had higher *M*_n_ values with narrower *M*_w_/*M*_n_ than the corresponding prepolymer irrespective of the amount of 1-octene added. These results indicate that the block copolymerization proceeded. The *M*_n_ values of resulting copolymers, however, decreased with an increase in 1-octene concentration in feed (Runs 1 and 2). The similar phenomenon was observed in copolymerization of propene and 1,7-octadiene with *C*_2_-symmetric *rac*-Me_2_Si[Ind]_2_ZrCl_2_ system (non-living catalyst system) [27].

**Figure 2:**
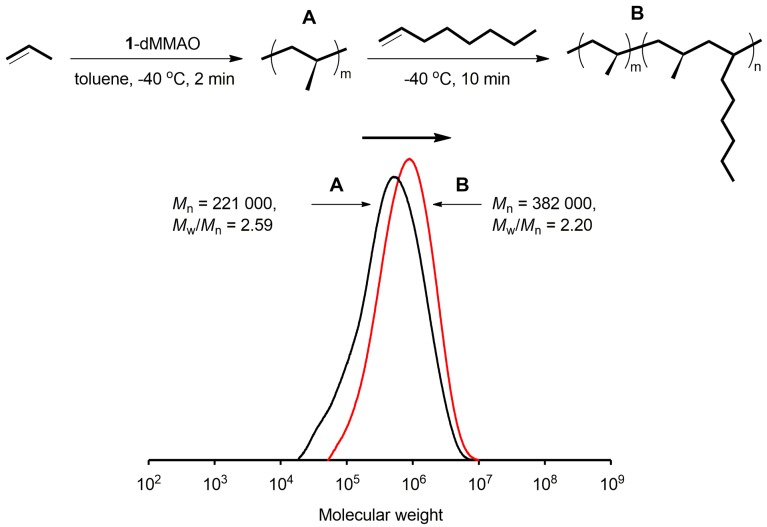
GPC curves of pre- and block-polymers obtained with **1**-dMMAO.

Figure 2 illustrates the GPC curves of the pre- and block-polymer obtained (Table 4, Run 1), where the GPC curve clearly shifted to the higher molecular weight region after the addition of 1-octene, indicating the living nature of the copolymerization.

**Figure 3:**
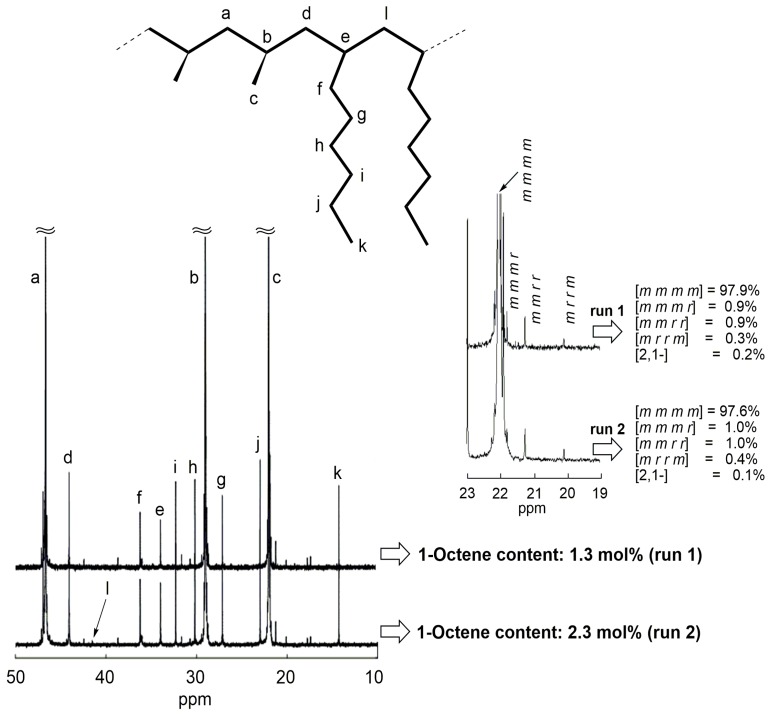
^13^C{^1^H}-NMR spectra of block copolymers and their expanded spectra of methyl regions obtained with **1**-dMMAO (Table 4, Runs 1 and 2).

The ^13^C{^1^H}-NMR spectra of the copolymers (Table 4, Runs 1 and 2) are shown in Figure 3, where the resonances assignable to the methylene carbon of the propene–propene sequence and that of propene-1-octene sequence appear at 47.1 and 44.3 ppm, respectively [28,29]. The block copolymers showed the melting point (*T*_m_ ≥ 150 °C, *mmmm* ≈ 98%) that corresponds to the crystalline *iso*-PP sequence. The ^13^C{^1^H}-NMR spectrum of the copolymer with high 1-octene feed (Table 4, Run 2) exhibited a resonance at 41.5 ppm, indicating the presence of 1-octene–1-octene dyad [28,29]. The 1-octene content values (OC cont.) determined by ^13^C{^1^H}-NMR are shown in Table 4, which indicates that the 1-octene content can be controlled with 1-octene concentration in feed.

## 3. Experimental Section

### 3.1. General Remarks

All operations were carried out under nitrogen atmosphere using standard Schlenk techniques. *rac*-Me_2_Si(2-Me-Benz(e)-Ind)_2_ZrCl_2_ (**1**) and *rac*-SiMe_2_(2-Me-4-Ph-1-Ind)_2_ZrCl_2_ (**2**) were obtained from the Boulder Scientific Company (Longmont, CO, USA). The toluene solution of modified methylaluminoxane (MMAO) donated from Tosoh-Finechem Co. Ltd. (Shunan, Yamaguchi, Japan). Trialkylaluminum-free MMAO (dMMAO) was prepared from the toluene solution of MMAO as reported previously [21]. Research grade propene (Takachiho Chemicals Industrial Co., Machida, Tokyo, Japan) was purified by passing it through columns of NaOH, P_2_O_5_, and molecular sieves 3A, and then by bubbling it through a NaAlH_2_Et_2_/1,2,3,4-tetrahydronaphthalene solution. 1-Octene (Tokyo Chemical Industries Co. Ltd., Chuo, Tokyo, Japan) and solvents were dried over calcium hydride and freshly distilled before use.

### 3.2. Polymerization Procedure 

Polymerization was performed in a 100 mL glass reactor equipped with a magnetic stirrer and carried out as follows. Under a nitrogen gas flow, the reactor was charged with a prescribed volume of solvent (heptane or toluene), and the reactor was then kept in a water bath of 0 °C or a dry ice–methanol bath of −40 °C. When polymerization was conducted at 0 °C, the solvent was saturated under an atmospheric pressure of propene. On the other hand, when polymerization was conducted at −40 °C, a certain amount of propene measured by a gas flow meter was dissolved in the solvent. Polymerization and block copolymerization were started by adding the solution of the zirconocene complex and dMMAO, which had been aged by the following procedure. After the activator and the zirconocene were dissolved in 5 mL of heptane or toluene, the solution was stirring for 5 min at room temperature and the polymerization temperature, respectively. Polymerization was conducted for prescribed time and terminated by adding acidic methanol. Block copolymerization of propene with 1-octene was conducted by the following procedure. After homo-polymerization of propene (3.5 g of propene in 30 mL of toluene) was conducted with **1**-dMMAO for 2 min, 1-octene (1.7 g or 3.4 g) was added and the copolymerization was successively conducted for 10 min. The polymerization was terminated by adding acidic methanol. The polymers obtained were adequately washed with methanol and dried under vacuum at 60 °C for 6 h. 

### 3.3. Analytical Procedure 

Molecular weights (*M*_n_) and molecular weight distributions (*M*_w_/*M*_n_) of polymers obtained were determined by gel permeation chromatography with a Waters 150CV (Waters Corp., Milford, MA, USA) at 140 °C using *o*-dichlorobenzene as a solvent. As parameters for universal calibration, K = 7.36 × 10^−5^ and α = 0.75 (for polystyrene standards) and K = 1.03 × 10^−4^ and α = 0.78 (for PP samples) were employed. Molecular weights and molecular weight distributions of block copolymers were determined using polystyrene standards without universal calibration. ^13^C{^1^H}-NMR spectra of polymers were measured at 120 °C on a JEOL GX 500 spectrometer (JEOL Ltd., Akishima, Tokyo, Japan) operated at 125.65 MHz in the pulse Fourier transform mode. In the ^13^C{^1^H}-NMR measurements, the pulse angle was 45° and about 10,000 scans were accumulated in a pulse repetition of 5.0 s. Sample solution was prepared in 1,1,2,2-tetrachloroethane-*d*_2_ up to 10 wt %. The central peak of tetrachloroethane-*d*_2_ (74.47 ppm) were used as an internal reference for ^13^C{^1^H}-NMR spectra. Differential scanning calorimetry measurements were made on a Seiko DSC-220 (Seiko Instruments Inc., Chiba, Japan). Polymer samples (ca. 5 mg) were encapsulated in aluminum pans, preheated at 200 °C for 5 min, and scanned at 10 °C/min.

## 4. Conclusions

In conclusions, a highly *iso*-specific quasi-living polymerization of propene with fast propagation was achieved at −40 °C by using a *C*_2_-symmetric zirconocene **1** activated by dMMAO. The catalyst was applied to the block copolymerization of propene and 1-octene, affording a copolymer containing a highly *iso*-PP block.

## Figures and Tables

**Table 1:**
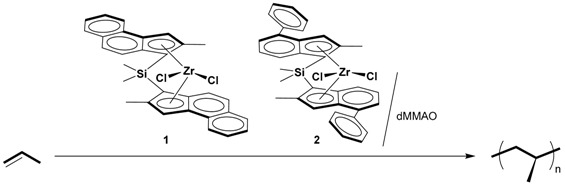
Propene polymerization with **1** and **2** activated by dMMAO ^a^.

Run	Complex	Solvent	[Al]/[Zr]	Time (min)	A ^b^	*M*_n_^c^ (× 10^4^)	*M*_w_/*M*_n_^c^	*N* ^d^
1	**1**	toluene	800	2	5400	18.0	2.22	1.00
2	**2**	toluene	800	5	3100	12.3	2.35	2.10
3	**1**	toluene	400	2	5000	16.4	3.08	1.00
4	**2**	toluene	400	5	2100	13.7	2.29	1.20
5	**1**	toluene	300	2	5000	23.8	2.33	0.70
6	**1**	heptane	300	10	300	44.9	2.71	0.11
7	**1**	toluene	100	2	230	36.6	2.26	0.02

^a^ Polymerization conditions: total volume = 30 mL; [Zr] = 10 μmol; propylene = 1 atm; temp. = 0 °C. ^b^ Activity in kg-PP·mol-Zr^−1^·h^−1^. ^c^ Determined by GPC using universal calibration. ^d^ Number of polymer chain calculated from yield and *M*_n_.

**Table 2:** Propene polymerization with **1**-dMMAO in heptane ^a^.

Run	Propylene (g)	Time (min)	Yield (%)	*M*_n_^b^ (×10^4^)	*M_w_/M_n_* ^b^	*N* ^c^ (μmol)
1	0.63	60	93	21.3	1.91	3
2	1.26	60	91	20.7	2.19	6
3	0.63 + 0.63	60 + 60	93	17.3	2.12	7

^a^ Polymerization conditions: total volume = 30 mL; [Zr] = 5 μmol; [Al]/[Zr] = 600; temp. = 0 °C. ^b^ Determined by GPC using universal calibration. ^c^ Number of polymer chain calculated from yield and *M*_n_.

**Table 3:** Propene polymerization with **1**-dMMAO using the sampling method ^a^.

Run	Solvent	Time (min)	*M*_n_^b^ (×10^4^)	*M*_w_*/M*_n_^b^ (×10^4^)
1	heptane	5	28.1	2.74
2	heptane	10	41.7	2.98
3	heptane	15	70.4	2.37
4	toluene	2	20.8	2.62
5	toluene	5	29.1	2.21
6	toluene	10	45.2	2.28

^a^ Polymerization conditions: solvent = 30 mL; [Zr] = 5 μmol; [Al]/[Zr] = 600; propene = 1 atm; temp. = −40 °C. ^b^ Determined by GPC using universal calibration.

**Table 4:** Block copolymerization of propene and 1-octene with **1**-dMMAO in toluene ^a^.

Run	1-Octene (mol %)	Time (min)	*M*_n_^b^ (×10^4^)	*M*_w_/*M*_n_^b^	OC Cont.^c^ (mol %)	*T*_m_^d^ (°C)
Prepolymer ^e^	-	-	22.1	2.59		
1	5	10	38.2	2.20	1.3	150
Prepolymer ^e^	-	-	26.6	2.48		
2	10	10	30.5	2.40	2.3	151

^a^ Polymerization conditions: total volume = 30 mL; [Zr] = 5 μmol; [Al]/[Zr] = 600; temp. = −40 °C. ^b^ Determined by GPC using universal calibration. ^c^ Determined by ^13^C NMR. ^d^ Determined by DSC. ^e^ Propene = 3.5 g; temp. = −40 °C; time = 2 min.

## Supplementary Materials

Supplementary materials can be accessed online.
