# Supplementary material for: Quasi-Living Polymerization of Propene with an Isotactic-Specific Zirconocene Catalyst
Source: Molecules. 2017 May 2;22(5):725. doi: 10.3390/molecules22050725 (PMC6154675; doi:10.3390/molecules22050725)
Supplement: Supplementary file 1 [file molecules-22-00725-s001.pdf]

Supporting Information

Quasi-Living Polymerization of Propene with an Isotactic-Specific Zirconocene Catalyst

Kei Nishii* [1]*, Miyuki Murase* [2] *and Takeshi Shiono* [3,]*

[1] Department of Materials Chemistry and Bioengineering, National Institute of Technology, Oyama College, 771 Nakakuki, Oyama, Tochigi 323-0806 Japan; k.nishii@oyama-ct.ac.jp

[2] Japan Polypropylene Corporation Polymerization Technical Center 1 Toho-cho, Yokkaichi, Mie, 510-0848, Japan (present affiliation)

[3] Department of Applied Chemistry, Graduate School of Engineering, Hiroshima University, Higashi-Hiroshima 739-8527, Japan; tshiono@hiroshima-u.ac.jp

*****Correspondence: tshiono@hiroshima-u.ac.jp; Tel.: +81-(0)82-424-7730

We roughly evaluated the rate constants of propagation (k_p) and chain transfer (k_{tr}) by eq. (4) reported by Keii et al. using the data in **Table 3**, although [M] is not constant in our conditions: Keii, T.; Terano, M.; Kimura, K.; Ishii, K. Makromol. Chem., Rapid Commun. 1987, 8, 583-587. b Keii, T.; Terano, M.; Kimura, K.; Ishii, K. In Transition Metals and Organometallics as Catalysts for Olefins Polymerization; Kaminsky, W., Sinn, H., Eds.; Springer-Verlag: Berlin, 1988; pp 3-12.

$$M_n = \frac{M_0 k_p [M][C^*]t}{[C^*] + k_{tr}[C^*]t} \quad (1)$$

$$\frac{M_0}{M_n} = \frac{k_{tr}}{k_p[M]} + \frac{1}{k_p[M]} \times \frac{1}{t} \quad (2)$$

$$P_n = \frac{M_n}{M_0} \quad (3)$$

$$\frac{1}{P_n} = \frac{k_{tr}}{k_p[M]} + \frac{1}{k_p[M]} \times \frac{1}{t} \quad (4)$$

where P_n is the number-average degree of polymerization, M_0 the molar mass of the monomer, [C*] number of active species, t polymerization time, [M] the monomer concentration, k_p the propagation rate constant, and k_{tr} the chain transfer rate constant.

By applying eq 4 to the data of **Table 3** (**Figure S1 and S2**), we obtained the k_p and k_{tr} values as shown in **Table S1**. Busico et al. reported a quasi-living propene polymerization using a N^N-chelating bis(phenoxy)Zr complex, where $\boldsymbol{k_p}$ **= 0.045** $L^{-1}\cdot mol^{-1}\cdot s^{-1}$ and $\boldsymbol{k_{tr}}$ **= 6 × 10**$^{\mathbf{-4}}$ s^{-1} (Busico, V.; Cipullo, R.; Fraldi, N.; Ronca, S.; Togrou, M. *Macromolecules* **2003**, *36*, 3806–3808.).

The $\boldsymbol{k_{tr}}$ values (**10 × 10**$^{\mathbf{-7}}$ s^{-1} and **13 × 10**$^{\mathbf{-7}}$ s^{-1}) of the present system are three orders of magnitude smaller than that of Busico's system. In addition, the $\boldsymbol{k_p}$ values (**22** $L^{-1}\cdot mol^{-1}\cdot s^{-1}$ and **9.5** $L^{-1}\cdot mol^{-1}\cdot s^{-1}$) are three orders of magnitude greater than that of Busico's system.

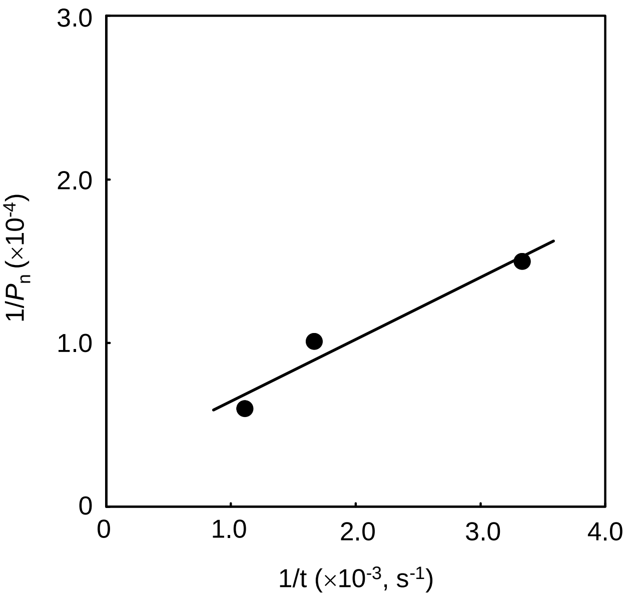

Figure S1. Plots of $1/P_n$ vs 1/t on Table 3 (heptane, [Al]/[Zr] = 300).

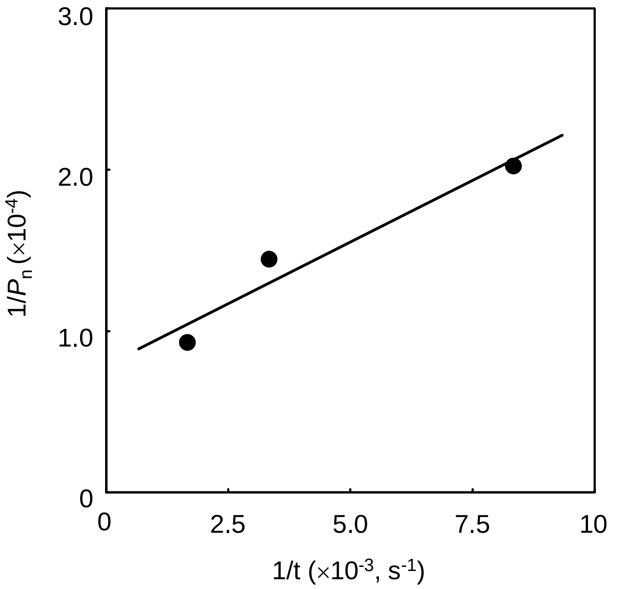

Figure S2. Plots of $1/P_n$ vs 1/t on Table 3 (toluene, [Al]/[Zr] = 300).

Table S1. The kinetic results obtained at various polymerization conditions.

runs	Solvent, [Al]/[Zr]	k_p[a] ($L^{-1}\cdot mol^{-1}\cdot s^{-1}$)	k_{tr}[a] ($\times 10^{-7}$, s^{-1})
1--3	Heptane, 300	9.5	10
4--6	Toluene, 300	22	13

[a] Approximate values obtained by means of Eq. (4).
